# Human infections with *Dermatophilus* sp.: travel-associated first case and a local cluster in the context of emerging European outbreaks, Basel, Switzerland, 2025 to 2026

**DOI:** 10.2807/1560-7917.ES.2026.31.29.2600552

**Published:** 2026-07-23

**Authors:** Peter Michael Keller, Roberta Di Nicola, Lucija Marković, Maximilian Köster, Claudia Lang, Marie-Thérèse Ruf, Christopher M Field, Sarah Tschudin-Sutter, Alexander Navarini, Maja Weisser

**Affiliations:** 1 Clinical Bacteriology/Mycology, University Hospital Basel, Basel, Switzerland; 2 Division of Infectious Diseases, University Hospital Basel, Basel, Switzerland; 3 Department of Dermatology, University Hospital of Basel, Basel, Switzerland; 4 Swiss TPH, Allschwil, Switzerland; 5 University of Basel, Basel, Switzerland; 6 Department of Laboratory Medicine, Bioinformatics Unit, University Hospital Basel, Basel, Switzerland; * These authors contributed equally to this work and share last authorship.

**Keywords:** *Dermatophilus congolensis*, human dermatophilosis, skin infection, sexually transmitted infections, outbreak investigation, whole-genome sequencing, Europe

## Abstract

We report six human *Dermatophilus* infections diagnosed in Basel between May 2025 and March 2026. The travel-associated first case developed a pustular rash and conjunctivitis after a stay in Thailand. A second imported case occurred after travel to Indonesia. Four subsequent locally acquired cases presented with genital or inguinal folliculitis-like lesions without reported animal contact. Whole-genome sequencing placed all isolates in a monophyletic cluster within the *D. congolensis*_A lineage, supporting possible human-to-human transmission. Enhanced surveillance and molecular diagnostics are warranted.

Since late 2025, several European countries have documented clusters of human dermatophilosis. Many cases occurred among men who have sex with men (MSM) and showed genomic linkage, suggesting human-to-human transmission, potentially associated with close skin-to-skin contact including during sexual or martial arts activities [1-3]. In June 2026 the European Centre for Disease Prevention and Control (ECDC) published a rapid risk assessment (RRA) on these multi-country clusters [4].

Here we report six cases of human *Dermatophilus congolensis*_A infection diagnosed at the University Hospital Basel, Switzerland, between October 2025 and June 2026. This retrospective review of patient records was initiated following the publication of the ECDC RRA on emerging human infections with *D. congolensis* in Europe. We describe clinical features, laboratory confirmation by whole-genome sequencing (WGS), phylogenetic relatedness of the isolates, and discuss implications for surveillance and public health.

## Case series and clinical presentation

We retrospectively identified all laboratory-confirmed *Dermatophilus* sp. infections at our institution from January 2025 to June 2026. Clinical, demographic, travel and exposure history were extracted from electronic medical records. A standardised case report form captured age, sex, clinical presentation, material sampled, comorbidities, sexual history, animal contact, travel, treatment and outcome. 

We identified six cases (Table). All patients were male (median age: 41 years; range: 25–59). The first case (Case 1) identified was a travel-associated infection in a man in his 40s who developed a pruritic pustular rash on the trunk, face and scalp as well as bilateral conjunctivitis approximately 1 week after returning from a Muay Thai martial arts training camp in Thailand (Khao Lak) in May 2025. Case 1 reported two mosquito bites and had direct animal contact (touching an elephant). The second detection was an imported travel-associated case (Case 2) returning from Indonesia and hereafter, we identified four cases locally acquired in Switzerland (Cases 3–6), with a temporal gap of 5 months between the imported and the autochthonous cases (Table). Cases 3–6 presented predominantly with genital or inguinal folliculitis-like lesions, some accompanied by dysuria. No case reported fever or systemic symptoms at presentation. Cases 1 and 4–6 had negative comprehensive sexually transmitted infections (STI) screening; Case 3 had serological evidence consistent with a past resolved or treated syphilis infection. The treatment responses were broadly consistent with the reported cases [1-4]. Cases 1 and 2 (imported cases) received systemic amoxicillin/clavulanic acid plus topical therapy and improved rapidly, while the Cases 3–6 (autochthonous cases) were successfully managed with topical antiseptics alone (betaine/polyhexanide and chlorhexidine digluconate), resulting in recovery or marked improvement in all cases.

**Table:** Demographic, clinical and laboratory characteristics of human *Dermatophilus congolensis*_A cases, Basel, Switzerland, May 2025–March 2026 (n = 6)

Case detection	Age range (years)/sex	Travel/ exposure^a^	Clinical presentation^b^	Key diagnostics	Therapy and outcome	WGS/phylogeny
1 (May 2025)	40–50 M	Thailand (Muay Thai martial arts camp)	Pustular rash (trunk, face, scalp), conjunctivitis	VZV/MPOX/measles negative; *D. congolensis*_A. (WGS; GTDB-Tk)	Amoxicillin/clavulanic acid 625 mg/125 mg 3 × daily for 7 days; topical therapy; improved	2.62 Mbp; 95.24% complete; 0% contamination; reference for phylogeny
2 (Jul 2025)	20–30 M	Indonesia (barefoot beach)	Disseminated pustules (face, extremities; impetigo-like)	*D. congolensis*_A (WGS)	Amoxicillin/clavulanic acid 625 mg/125 mg 3 × daily for 10 days; topical therapy; resolved	2.64 Mbp; 95.24% complete; 0% contamination
3 (Dec 2025)	50–60 M	None reported	Genital folliculitis (inguinal)	*D. congolensis*_A (WGS); STI screen^b^ negative (serological evidence of past treated/resolved syphilis)^c^	Topical only (betaine/polyhexanide and chlorhexidine digluconate); recovered	2.64 Mbp; 95.05% complete; 0.23% contamination
4 (Jan 2026)	50–60 M	None reported	Genital eczema; dysuria; history of multiple skin lesions	*D. congolensis*_A (WGS and shotgun metagenomics on biopsy); biopsy: spongiotic dermatitis and visible bacteria (microbial eczema)	Topical (betaine/polyhexanide and chlorhexidine digluconate); recovered	2.65 Mbp; 95.24% complete; 0.55% contamination
5 (Jan 2026)	20–30 M	None reported	Genital folliculitis; dysuria	*D. congolensis*_A (WGS); STI screen negative	Topical (betaine/polyhexanide and chlorhexidine digluconate); recovered	2.63 Mbp; 95.24% complete; 0% contamination
6 (Feb/Mar 2026)	50–60 M	None reported	New folliculitis on background of chronic skin lesions (condylomata, molluscum, skin tags)	*D. congolensis*_A (WGS)	Topical (betaine/polyhexanide and chlorhexidine digluconate); recovered	2.87 Mbp; 95.51% complete; 11.72% contamination

GTDB-Tk: Genome Taxonomy Database Toolkit; M: male; STI: sexually transmitted infection; VZV: varicella zoster virus; WGS: whole genome sequencing.

^a^ Sexual history was assessed during routine care where feasible but not systematically documented. No systematic data on attendance at saunas or sex-on-premises venues wer available.

^b^ Dates refer to the month of initial clinical presentation at the hospital, when key clinical information was documented.

^c^ STI screen: testing for HIV, hepatitis B and C virus, herpes simplex virus-1/2, syphilis and mpox, as well as nucleic acid amplification test for *Chlamydia trachomatis* and *Neisseria gonorrhoeae.*

## Whole genome sequencing and phylogenetic analysis

Clinical specimens (superficial lesion swabs) were cultured on Columbia blood agar and chocolate agar at 37 °C in a 5% CO_2_ atmosphere. Bacterial identification was performed by MALDI-TOF mass spectrometry (MS; Bruker Daltonics, Bremen, Germany) and, when scores were inconclusive, by 16S rRNA gene sequencing and WGS. Illumina paired-end reads were quality controlled with BBDuk from BBTools v39.13 [5]. Adapter sequences were removed by right-end trimming, PhiX reads were filtered, and reads were quality trimmed (Phred score cut-off of 14 and mean read-quality cutoff of 20). We excluded reads shorter than 45 bp or containing ambiguous bases. Genomes were assembled with SKESA v2.4.0 [6]. Snippy v4.6.0 [7] was used to call variants for each sample against the bacterial genome of Case 1 with downsampling to a maximum of 100-fold coverage and option --minfrac 0.7, before calculating a core genome of 2,617,836 bp containing 11 variant sites. Phylogeny was constructed with PhyML v3.3.20250515 [8] and visualised in R 4.4.2 with the ape v5.8–1 and phytools v2.5–2 packages [9,10]. Genomes were classified with the Genome Taxonomy Database Toolkit (GTDB-Tk) v2.4.0 [11] with data release 226 and checked for completeness and contamination with CheckM v1.2.4 [12]. 

All six isolates were classified as *D. congolensis*_A, a genomic lineage distinct from the *D. congolensis* type strain (average nucleotide identity [ANI] < 95%). We have appended the ANI values as well as the single nucleotide polymorphism (SNP) distance matrix of the cases’ isolates in the Supplementary Tables. The genomes contained > 95% of marker genes for the order *Actinomycetales* by CheckM. Core genome SNP phylogeny with previously published cases from Lyon and Paris [2], and the 43 genome sequences available in NCBI GenBank showed a clear split into two clusters, placing our isolates far from the type and reference strains, and instead with the French isolates. The core genome SNP-based phylogenetic tree using NCTC 13039 type strain isolated from cattle scabs (1956, Zambia) as reference has been appended in Supplementary Figure S1. We recalculated a second phylogeny using the closest published genome as reference, strain NCTC 7915 isolated from sheep hoof (1949, London). This phylogenetic tree has been appended in Supplementary Figure S2 and showed that our isolates mixed with the French isolates but were distinct from the reference by > 13,000 SNPs. As the reference was still very distant from our case genomes, we calculated a third phylogeny, with the earliest case (Basel Case 1, May 2025) as the reference sequence; this showed parsimonious temporal and geographical clusters, with a maximum distance between isolates of only 5 SNPs and a genomically identical cluster of the sequences from Basel Cases 3 and 4 together with those from French Cases 4, 5 and 6 (Figure).

**Figure:**
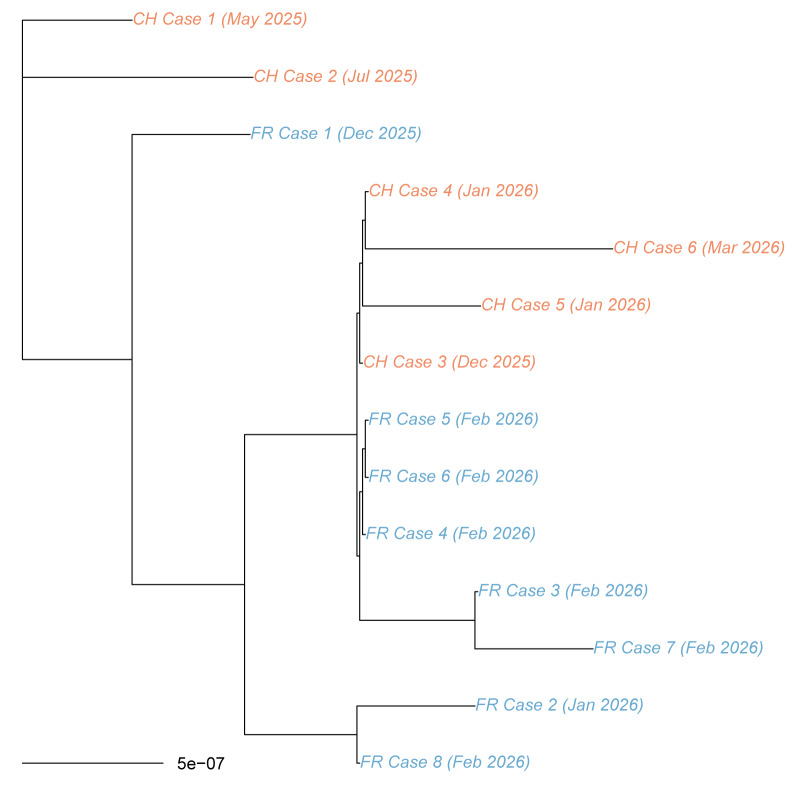
Core genome SNP-based phylogenetic tree of *Dermatophilus congolensis*_A isolates (reference strain CH Case 1)

Isolates are labelled with case dates and coloured by country: FR: France; CH: Switzerland. The scale bar represents 5 × 10^−7^ substitutions/site.

## Discussion

This report describes a cluster of six human infections with *D. congolensis*_A in Basel, Switzerland, occurring between May 2025 and June 2026. *Dermatophilus congolensis* is a Gram-positive, facultative anaerobic actinomycete that causes exudative dermatitis (dermatophilosis or 'rain rot') primarily in cattle, sheep, horses and other animals. The organism produces motile zoospores that invade damaged skin, leading to characteristic crusting lesions. Human infections are uncommon and usually zoonotic, resulting from direct contact with infected animals or contaminated fomites, especially in tropical and subtropical regions [13,14]. In 2021, de Lorenzi et al. reported a travel-associated human case linked to elephant riding in Thailand [15]. Together with recently published cases from Barcelona, Lyon, Paris and Stockholm [1-3], these findings add to the growing evidence of an emerging zoonotic pathogen causing human infections across Europe. 

Case 1 was travel-associated, with a history of close animal contact during a trip to a high-risk area, mirroring a previously reported travel-related case linked to elephant riding in Thailand [15]. This case could have been the primary case in this cluster; however, our retrospective review of clinical records did not comprise an in-depth investigation of epidemiological links and there were no indications of links between cases. Nevertheless, our observation points out travel to endemic or enzootic regions in South-East Asia as a potential source of introduction into Europe. Interestingly, the sequence obtained from Case 2, the second travel-associated case returned from Indonesia, clustered phylogenetically closely with that of Case 1 based on SNP analysis, despite different travel destinations. Subsequent cases, occurring from December 2025 onwards and more distant in time, formed a separate cluster that grouped closer to the published French isolates.

These genomic findings raise the possibility of limited local transmission events following introduction, although definitive proof of sustained human-to-human transmission remains elusive given the small number of cases and challenges in epidemiological tracing. Further surveillance and genomic studies including targeted sampling and bacterial WGS from animal reservoirs and human cases in endemic South-East Asia are warranted. With respect to possible relatedness of strains circulating in Thailand and Indonesia, publicly available genomic data for *D. congolensis*_A from Indonesia remain very limited. Although reports of dermatophilosis in animals exist in Indonesia and human cases have been linked to travel in Southeast Asia (including Thailand), strain-level resolution is currently insufficient to allow direct comparison [2,15]. The cases acquired in Switzerland (Cases 3–6) occurred without reported animal or travel exposure, coinciding temporally with the multi-country European clusters described by the ECDC and by national surveillance [1-4].

The phylogenetic analysis revealed that the locally acquired isolates from Basel formed a tightly related genetic cluster. Such tight clustering is consistent with either a single introduction event followed by local spread or repeated introductions of the same strain. The small SNP distances support recent common ancestry and argue against multiple independent zoonotic acquisitions. The clinical presentation (genital and inguinal predominance) and the wider European MSM context strongly suggest that close skin-to-skin contact such as during sexual or martial arts activities can facilitate spread, analogous to the outbreaks in Barcelona, Lyon/Paris, Stockholm and Trondheim [1-3].

A limitation of this study is that the data were based solely on information obtained during routine individual patient care. No systematic contact tracing or detailed interviews regarding risk factors, including sexual behaviour, were conducted to determine precise transmission routes, as a more detailed epidemiological investigation was deemed disproportionate given the generally mild and often self-limiting nature of the disease and the priority of protecting patient privacy. In addition, although recently reported Norwegian cases associated with martial arts activities and travel to Thailand are highly relevant in the context of Case 1, they could not be included in the phylogenetic analysis because the corresponding sequence data were not publicly available.

Laboratory diagnosis benefited from WGS, which provided both species confirmation and phylogenetic context. Routine MALDI-TOF MS occasionally yielded borderline scores, and conventional biochemical tests are unreliable for this organism [16-18]. We therefore recommend that any isolate morphologically suggestive of *Dermatophilus* sp. (Gram-positive branching rods, beta-haemolytic, dry, dented colonies) be referred for WGS, especially in the current epidemiological setting.

Public health implications are twofold. Firstly, clinicians should include *Dermatophilus* sp. in the differential diagnosis of folliculitis, pustular dermatitis or genital ulcers, particularly in travellers returning from tropical regions or in MSM presenting with compatible lesions. Secondly, the emergence of this pathogen in Europe calls for the rapid development and sharing of a core genome MLST (cgMLST) scheme to enable standardised, high-resolution surveillance across countries. Such a scheme would allow real-time tracking of introductions vs local transmission and guide targeted interventions.

## Conclusion

Human *Dermatophilus* infections should no longer be regarded solely as sporadic zoonoses. The Basel case series, comprising two travel-associated cases followed by four locally acquired infections, illustrates new importation routes and the potential for sustained local transmission. Heightened clinical awareness, prompt molecular confirmation by WGS, and international collaboration on typing schemes are essential to understand and contain this emerging public health concern.

## Data Availability

No datasets were generated beyond the clinical and microbiological data described in this report. Sequencing reads have been deposited in the European Nucleotide Archive project PRJEB115583 with accessions ERR17454709-12, ERR17454714 and ERR17454718.

## Supplementary Data

47000 SupplementaryTables

## Supplementary Data

173000 SupplementaryFigures
