# Supplementary material for: Human infections with Dermatophilus sp.: travel-associated first case and a local cluster in the context of emerging European outbreaks, Basel, Switzerland, 2025 to 2026
Source: Euro Surveill. 2026 Jul 23;31(29):2600552. doi: 10.2807/1560-7917.ES.2026.31.29.2600552 (PMC13402898; doi:10.2807/1560-7917.ES.2026.31.29.2600552)
Supplement: SupplementaryFigures [file 26-00552_KELLER_Supplementary_Figures.pdf]

Disclaimer: This supplementary material is hosted by Eurosurveillance as supporting information alongside the article "Human infections with *Dermatophilus* sp.: travel-associated index case and a local cluster in the context of emerging European outbreaks, Basel, Switzerland, 2025 to 2026", on behalf of the authors, who remain responsible for the accuracy and appropriateness of the content. The same standards for ethics, copyright, attributions and permissions as for the article apply. Supplements are not edited by Eurosurveillance and the journal is not responsible for the maintenance of any links or email addresses provided therein.

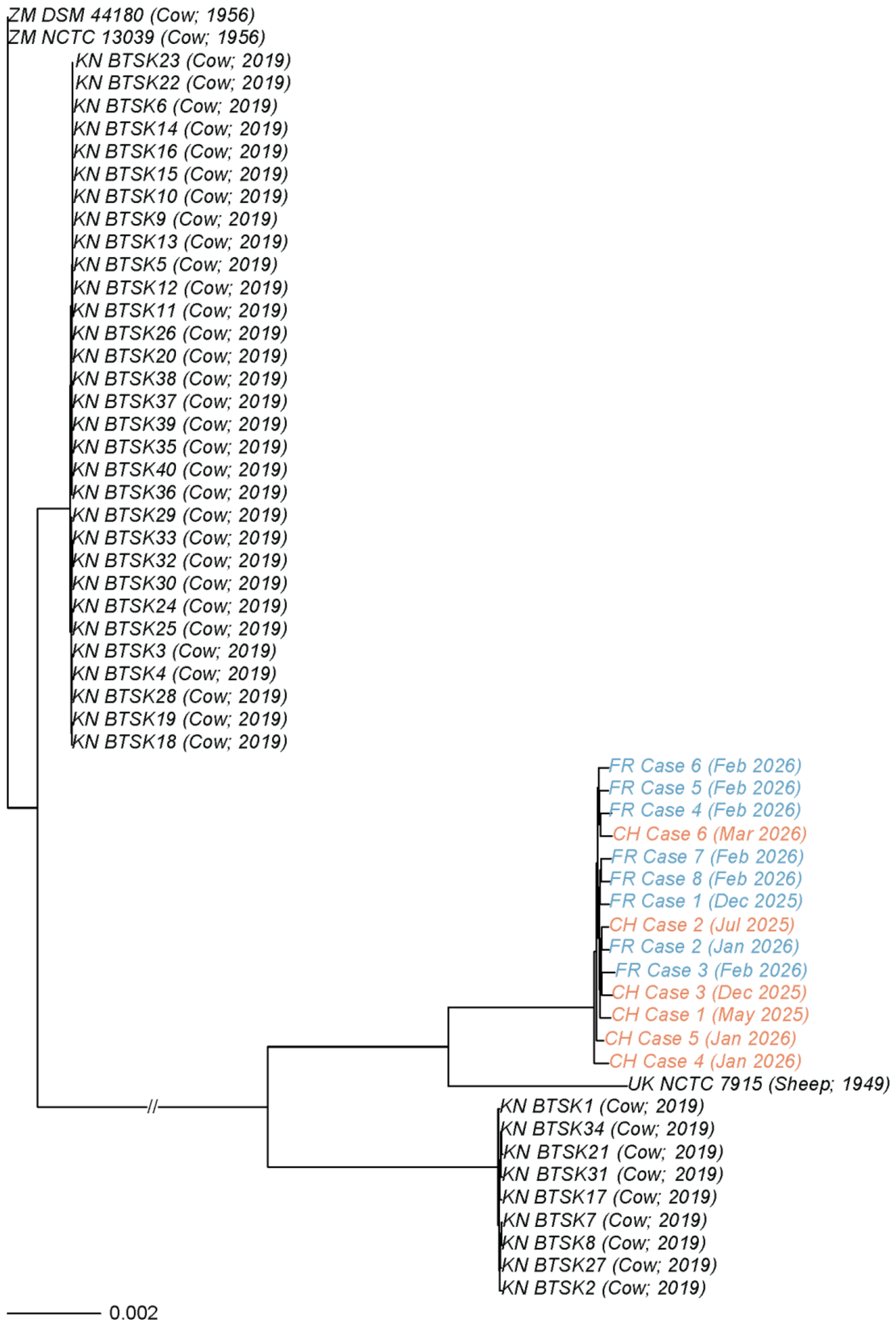

Supplementary Figure 1. Core genome SNP-based phylogenetic tree of *Dermatophilus congolensis*_A isolates. The tree was constructed using maximum-likelihood analysis with NCTC 13039 as reference strain. The scale bar represents 0.002 nucleotide substitutions per site. Sample origins are indicated by country codes (CH: Switzerland; FR: France; KN: St. Kitts and Nevis; UK: United Kingdom; ZM: Zambia). Cow isolates from a previous study are labelled with their original study identifier (https://doi.org/10.1128/MRA.00334-21).

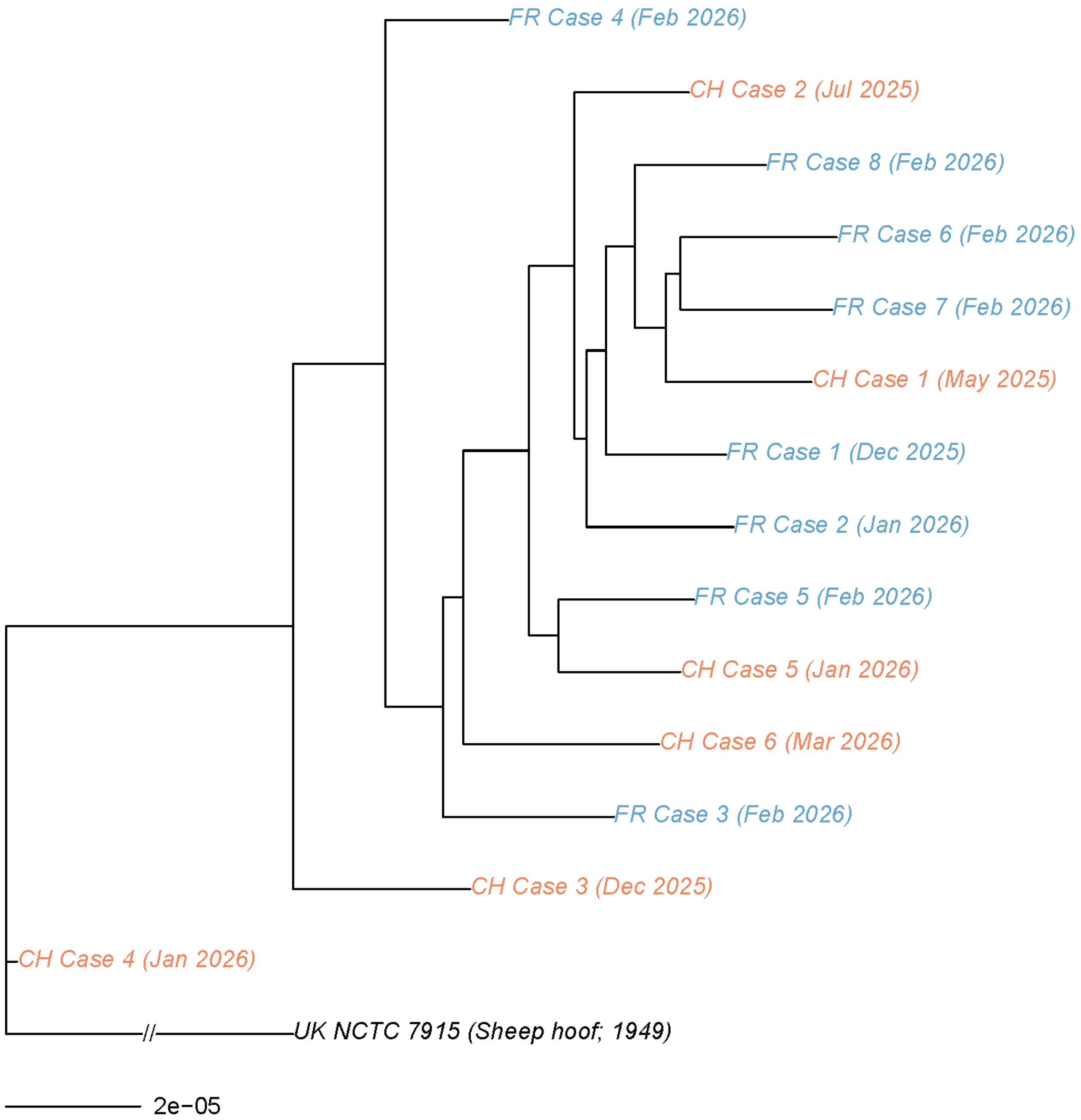

Supplementary Figure 2. Core genome SNP-based phylogenetic tree of *Dermatophilus congolensis*_A isolates. The tree was constructed using maximum-likelihood analysis. NCTC7915 (sheep hoof) was used as reference strain. The scale bar represents 2×10^{-5} nucleotide substitutions per site. Patient isolates are indicated by country code and color; CH represents Switzerland isolates in red, and FR previously published recent human isolates from France (Paris, Lyon) in blue. The branch between reference and the other samples was truncated for visual purposes only.
